# Zika virus: an overview update

**DOI:** 10.1097/COH.0000000000000926

**Published:** 2025-03-17

**Authors:** Hanna K. de Jong, Martin P. Grobusch

**Affiliations:** a Center of Tropical Medicine and Travel Medicine, Department of Infectious Diseases, Amsterdam UMC location University of Amsterdam, Amsterdam Institute for Immunology and Infectious Diseases, Amsterdam Public Health - Global Health, Amsterdam, The Netherlands; b Masanga Medical Research Unit (MMRU), Masanga, Sierra Leone; c Institute of Tropical Medicine, German Centre for Infection Research (DZIF), University of Tübingen, Tübingen, Germany; d Centre de Recherches Médicales en Lambaréné (CERMEL), Lambaréné, Gabon; e Institute of Infectious Diseases and Molecular Medicine (IDM), University of Cape Town, Cape Town, South Africa

**Keywords:** antivirals, arboviral disease, microcephaly, mAbs, prophylaxis, travellers, tropical infectious disease, Zika virus

## Abstract

**Purpose of review:**

Although cases of Zika virus disease (ZVD) have declined globally since 2017, new outbreaks have been reported, such as in Asia in 2024. As there is no vaccine or treatment available to date, both vaccines and mAbs neutralizing Zika virus would be of great interest, especially for pregnant women and immunocompromised patients such as those living with HIV. This review focuses on new insights regarding ZVD in the last two years and summarizes the key literature on global epidemiology, transmission, diagnostics, clinical features, preventive measures, and treatment options.

**Recent findings:**

At the time of writing, ZVD is endemic across tropical and subtropical regions of the world, with the highest risk of infection in Latin America and the Caribbean, but no significant peaks in outbreak activity across endemic regions. There are ongoing efforts to further investigate the clinical and epidemiological long-term sequelae of the large outbreak in the Americas 2015–2018; further refinement of diagnostic tools to improve specificity in view of significant cross-reactivity potential, particularly with dengue virus. Multiple vaccines are in different clinical development stages; however, phase 3 trials are awaiting the next epidemic.

**Summary:**

While there is no current major zika virus outbreak, progress has been made in the epidemiological work-up of clinical-epidemiological data, refinement of diagnostic tools, and mainly preventive (vaccines) rather than curative (drugs) tools.

## INTRODUCTION

Zika virus disease (ZVD) cases have been reported since the 1950 s in several African countries and since 1966 have been detected on the Asian continent, but from 2015 onwards, ZVD swept through the Americas reporting its peak in more than 500 000 infected cases [1]. The risk of severe infection is low, and in relation to overall patient numbers, few deaths in adults have been reported. However, its impact was greater than expected because of an increase in babies born with microcephaly during the epidemic peak, a surge in Guillain-Barré Syndrome case numbers, an extremely rare but live-threatening immune-induced thrombocytopenia, and overall, a risk of sexual transmission in the viraemic phase. Although cases of ZVD have declined globally since 2017, newer but smaller outbreaks have been reported, such as in Thailand and India in 2024. As there is no vaccine or treatment available yet, mAbs neutralizing Zika virus would be of great interest, especially for pregnant women and immunocompromised patients. This review focuses on new insights regarding ZVD in the last 2 years and summarizes the key literature on global epidemiology, diagnostics and clinical features, pathophysiology, preventive measures, and treatment options. 

**Box 1:**
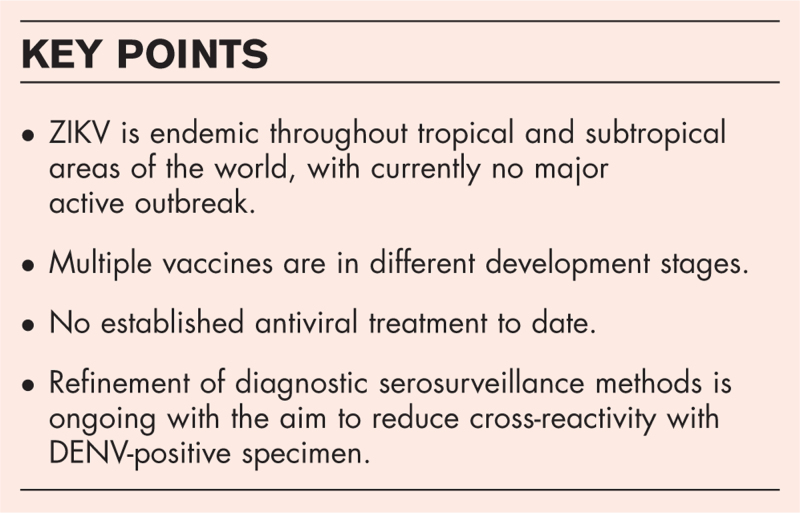
no caption available

## MATERIALS AND METHODS

For this scoping review, articles with the search term ZVD were screened (*n* = 2277) using the publicly available PubMed database (2277 results). Articles published between 2022 and 2024 (up to October 9th) and deemed relevant to our topic of focus were included in this review. Regarding clinical trials, the registry clinicaltrial.gov was searched by the authors (October 10th, 2024), and treatment strategies targeting ZVD undergoing phases 1, 2, 3, and 4 clinical trials (Table 1) were included in this review. Additional references were identified through a manual search of the reference lists of the identified publications.

**Table 1:** Zika virus vaccines in clinical development stages

Year of publication/clinical phase	Vaccine name and phase	Platform	Number of participants enrolled	Outcome
2018, phase 1 [27]	purified formalin-inactivated Zika virus vaccine (ZPIV) – Phase 1	Whole virus inactivated vaccine	68, of which 55 received the vaccine	Mild-to-moderate AE, At day 57, 52 (92%) of vaccine recipients had seroconverted (micro-neutralization titres ≥1 : 10), with peak geometric mean titres seen at day 43 and exceeding protective thresholds seen in animal studies.
2018, phase 1 [32]	VRC5288 plasmid VRC5288 (Zika virus and Japanese encephalitis virus chimera, VRC5283 (wild-type Zika virus), phase1	DNA vaccines	VRC 319 enrolled 80 participants (20 in each group), and VRC 320 enrolled 45 participants (15 in each group)	Mild-to-moderate AE. For VRC5283, 14 of 14 (100%) participants who received split-dose vaccinations by needle-free injection had detectable positive antibody responses, and the geometric mean titres of 304 was the highest across all groups in both trials.
2020, phase 1 [28]	purified formalin-inactivated Zika virus vaccine (ZPIV) – Phase 1	Whole virus inactivated vaccine	Participants were sequentially enrolled into one of three groups: ZPIV given at weeks 0 and 4 (standard regimen), weeks 0 and 2 (accelerated regimen), or week 0 alone (single-dose regimen). Enrolment of 12 participants per group (10 vaccine, 2 placebo)	Only mild-to-moderate AE were reported with a 52-week follow-up. ZPIV immunogenicity required two doses and was not durable. FU study 2023 [29] subjects were being primed with another flavivirus before immunization (YF or JEV), ZPIV was well tolerated in flavivirus naive and primed adults but immunogenicity varied significantly according to antecedent flavivirus vaccination status.
2021, phase 1 [34]	Ad26.ZIKV.001, a prophylactic ZIKV vaccine candidate	Vector-vaccine	Enrolment of 100 healthy volunteers, administered in one or two-dose regimens of 5 × 1010 or 1 × 1011 viral particles (vp), or placebo	No safety concerns. In both two-dose regimens, ZIKV nAbs peaked 14 days after the second vaccination and persisted for a year. A one-dose regimen induced 100% seroconversion and with titres persisting for at least 1 year. FU study 2024 [35]: vaccine tested in pregnant rhesus macaques showing the feasibility of vaccination against Zika during pregnancy.
2021, phase 1 [30]	TAK-426 is a purified, inactivated Zika virus vaccine with aluminium hydroxide as adjuvants	Inactivated whole virus vaccine	271 enrolled, (125 flavivirus-naive and 146 flavivirus-primed participants).	TAK-426 was well tolerated, with an acceptable safety profile, and was immunogenic in both flavivirus-naive and flavivirus-primed adults. The 10 μg TAK-426 dose was selected for further clinical development. FU study 2023 [31]: 2-year persistence of antibodies, compared to natural infection was similar.
2021, phase 1 [33]	GLS-5700, encoding the ZIKV premembrane and envelope proteins	DNA vaccine	Enrolment of 2 groups of 20 participants each	After 14 weeks, no SAEs were reported, nAbs developed in 62% of the samples on Vero-cell assay. On neuronal-cell assay, there was 90% inhibition of ZIKV infection in 70% of the serum samples and 50% inhibition in 95% of the samples.
2023, phase 1 [36]	mRNA-based Zika virus vaccines (mRNA-1325 and mRNA-1893)	mRNA vaccine	Enrolment of 90 participants in the mRNA-1325, and 120 participants in the mRNA study.	Both vaccines were generally well tolerated, but the mRNA-1325 vaccine elicited poor Zika virus-specific nAbs. On day 57, all evaluated mRNA-1893 dose levels induced robust Zika virus-specific nAb responses, independent of flavivirus serostatus, that persisted until month 13 FU study 2023: mRNA-1893 generated comparable neutralizing antibody titres to mRNA-1325 at 1/20th of the dose and provided complete protection from ZIKV challenge in nonhuman primates.

AE, adverse event; JEV, Japanese encephalitis virus; nAB, neutralizing antibody; SAE, serious adverse events; YF, yellow fever; ZIKV, zika virus.

## UPDATE ON GLOBAL EPIDEMIOLOGY

In 2019, Musso *et al.*[2] provided a comprehensive review of the epidemiological situation before, after, and during the epidemic spread of Zika virus (ZIKV) across the Pacific from 2007 onwards, and then throughout the tropical and subtropical Americas from 2015. Global trends and regional differences were recently summarized by Guo *et al.*[3]. *Aedes (Ae.) aegypti*, as the principal vector of ZVD, is found in 142 countries and territories worldwide, with ongoing transmission in 92 countries (as of May 2024) [4]. Against the backdrop of continued transmission with different intensities across tropical and subtropical regions of the world, there are currently no large outbreaks. For the time being, global incidence has steadily declined since 2007. A global seroprevalence analysis showed that the worldwide epidemiological situation was well reflected in those data; with an overall seroprevalence of 21% from 49 ZIKV-endemic countries and territories, it was highest in the Americas [39%; 95% confidence interval (95% CI) 16–26], in accordance with the recent epidemic [5]. With the offspring of women of childbearing age at particular risk for severe complications, Qin *et al*. [6] extracted annual incidence cases and ZIKV disease rates of women at reproductive age from the 2012 Global Burden of Disease (GBD) data, using relative percentage change in cases, and estimating the annual percentage change (EAPC) of incidence rates to quantify temporal trends. Peaking in 2016 at 174/100 000 population, the EAPC decrease from 2016 to 2021 was -52% at 3.06/100 000 population. The data indicate that women of reproductive age in Latin America and across the Caribbean continue to bear the highest risk of ZIKV infection compared to other world regions [6].

The economic burden of arboviral diseases is significant; one recent study in returning travellers to nonendemic countries calculated that among the nine ZVD patients in a cohort of 134 patients with malaria and dengue, chikungunya and ZVD disease, respondents experienced a disease-related loss of a median of 1500 USD (IQR 510–2625), respectively [7].

## TRANSMISSION RISK

Accoti *et al.*[8] showed that different larval microbiomes drive differences in the ZIKV infection rates in genetically diverse *Ae. aegypti*, demonstrating that interactions between the mosquito and its environment can influence ZIKV infection depending on the mosquito genotype, a finding that might be important for future transmission control strategies. The transmission risk and outbreak propensity depend on multiple factors. Several recent studies have examined the complex interplay between mosquitoes and host and vector microbiomes as factors influencing virus transmission. Sun *et al.*[9^▪^] demonstrated that *Enterobacter hormaechei*_B17, which forms a part of the midgut microbiome of both *Ae. aegypti* and *albopictus* mosquitoes produce sphingosine, which inhibits ZIKV infection *in vitro* by limiting viral membrane fusion, inhibits ZIKV infection *in vitro*. This mechanism may be exploited in future vector-control strategies. Not only the (bacterial) microbiome, but also mosquito-specific viruses might influence the ZIKV transmission capacity of individual mosquitoes, but also in terms of enhancing the arboviral transmission capacity. Spatiotemporal analyses of virus circulation in endemic urban areas have yielded a 200% increase in dengue virus (DENV) in *Ae. aegypti* mosquitoes harbouring both Phasi Charoen-like virus (PCLV) and Humaita Tubiacanga virus (HTV), a finding supported in a mouse model demonstrating enhanced transmission of DENV and ZIKV viruses to a vertebrate host by means of upregulation of a particular proviral host factor [10].

Climate change is expected to negatively affect many mosquito-borne diseases, which also applies to ZVD. Zika virus transmission occurs between temperatures of approximately 24°C and 34°C, peaking at 26°C–29°C [11]. Multiple mechanisms have been identified in favour of a projected expansion of intensity and geographical spread of ZIKV into, up to now, more temperate areas, including extended transmission seasons, changes in and expansion of vector habitats, reduced abundance of mosquito predators, and other factors such as potential decreases in control operations and other factors not directly related to climate change [11]. The current modelling data support a broadening of *Ae. albopictus* ecological niche in the Americas and Europe, calling for an increase in surveillance and mosquito control capacity [12].

## NOVEL DIAGNOSTIC TESTING APPROACHES AND NOVEL SURVEILLANCE APPROACHES

The most-commonly used diagnostic methods for acute ZIKV infections include blood and urine PCR, as well as sequential IgM and IgG detection from paired sera. Only IgG detection was used for surveillance purposes. Antigenic overlap, and thus high antibody cross-reactivity between DENV and ZIKV, renders serological testing for both acute and past ZIKV infections complicated. Pereira *et al.*[13] proposed a novel approach to overcome DENV/ZIKV cross-reactivity in serodiagnosis. The group designed, produced, and purified three multiepitope proteins, ZIKV-1, ZIKV-2, and ZIKV-3, which were used in an ELISA to detect ZIKV IgG. Sensitivities ranged from 66 to 95%, and specificities ranging from 84 to 97%, respectively. As no cross-reactivity was observed with DENV and CHIKV-positive sera, the method might yield potential for further development, particularly for seroprevalence study purposes. Another novel experimental approach to the same problem was reported by Castanha *et al.*[14^▪^], who identified a nonbiological molecule CZV1-1 from a small synthetic molecule library screening that binds specifically to ZIKV-IgG but not DENV-IgG. This method might prove to be valuable when it comes to avoiding or minimizing DENV-IgG cross-reactivity in future seroprevalence studies. Another novel approach aimed at overcoming the difficulty of distinguishing closely related viruses in arboviral serology is the identification of antibodies binding to overlapping peptides, or the lack thereof, from proteomes of almost 700 human and zoonotic arboviruses in high throughput and on epitope level by means of a programmable phage display platform (‘Arboscan’) [15].

Two recent studies put forth the practical application of a novel multiplex lateral flow immunoassay combined with IgM and IgG rapid diagnostic test panel (‘DPP Fever Panel ASIA’) [16], as well as a multiplex PCR panel (Biofire) [17] for the diagnosis of acute febrile illnesses. In a series of 300 patients with febrile illness from the Laos PDR. ZIKV IgM sensitivities from whole blood and serum samples were 100 and 75%, respectively (*n* = 8), with specificities of 77 and 99%, respectively. However, ZIKV IgG sensitivities were only 50 and 54% with specificities of 50 and 53% (*n* = 66), respectively. In a Spanish cohort of 455 returning travellers with acute fever, BioFire results were negative in both cases, which were NAAT positive, and in all 10 cases that were positive in composite RDT+NAAT+ serology testing, indicating the need for further investment in refining both molecular and serological testing of ZIKV versus other arboviral diseases.

Regarding the improvement of disease surveillance for the early recognition of outbreak activities or changes in epidemiology beyond outbreak situations, much recent attention has been drawn towards wastewater surveillance as an upcoming tool to be more systematically used for the detection and characterization of viruses with pandemic potential and beyond, including ZIKV [18]. Methodologically, targeted PCR amplification rather than metagenomic, nontargeted amplification is required because of the low concentrations of the target viral RNA among the overall abundance of RNA from different organisms [18].

## CLINICAL FEATURES, INCLUDING RISK FOR PEOPLE LIVING WITH HIV/AIDS

ZIKV disease has been associated with three groups of notable adverse outcomes, namely Guillain-Barré Syndrome (GBS), which is now considered to occur with approximately the same frequency of 2–3/10 000 clinical cases, comparable to *Campylobacter* spp. infections [2]: autoimmune thrombocytopenia, which is extremely rare but potentially life-threatening [19], particularly with microcephaly and other adverse neurological outcomes in in utero ZIKV-exposed newborns. With regards to the latter, large long-term prospective cohort studies set up during the large ZIKV disease outbreak sweeping through South and Central America in 2015 have reported recent updates, mainly from Brazil. Paixao *et al.*[20] reported 36-month follow-up data for an 11.5 million live-born paediatric population-based cohort study assessing mortality among children with and without congenital Zika syndrome. The mortality rate ratio among children with congenital Zika syndrome as compared to those without was 11.3 (95% CI 10.2–12.4). In a meta-analysis of 13 pooled Brazilian cohorts, the risk of adverse outcomes in offspring with PCR-confirmed prenatal ZIKV exposure was as high as 1548 ZIKV-exposed pregnancies, and miscarriage and stillbirth rates were 0.9 and 0.3%, respectively. The absolute risks for microcephaly and functional neurological abnormalities were 2.6 and 18.7%, respectively [21^▪^]. The risk was comparable across study sites and socioeconomic strata, indicating the absence of risk modifiers other than ZIKV. Of note, investigators from Nicaragua who assessed neurodevelopmental scores between normocephalic in utero ZIKV-exposed children and unexposed controls (*n*_total_ = 1091) did not identify any significant neurodevelopmental score difference between the two groups [22]. Some regional variations other than those due to different methodologies in patient assessment and analytic approach might apply; however, the Brazilian data might serve as a proxy due to their overwhelmingly large cohort sizes.

Previously published data suggest that no adverse outcomes increase in both iatrogenically immunocompromised patients and HIV-positive individuals. Existing data were summarized and confirmed in a recent nonsystematic review [23].

## UPDATE ON PREVENTIVE MEASURES AND TREATMENT OPTIONS

### Vaccines

During the heydays of the South American epidemic, the first clinical trials of vaccines targeting ZIKV were conducted. Vaccines in the clinical development stages targeting ZVD are summarized in Table 1. However, many more vaccines are in preclinical development [24], including vaccines developed for pregnant women [25] and a vaccine to be applied intranasally [26]. Focusing on the vaccines in the clinical pipeline thus far, only phase 1 studies in healthy volunteers have been published and these include: two whole-virus inactivated vaccines, two DNA vaccines, one mRNA vaccine, and one virus-vectored vaccine. The purified formalin-inactivated Zika virus vaccine (ZPIV) was reported to be well tolerated and immunogenic in humans for up to eight weeks in its first phase 1 trial [27]. In the second phase 1 trial, it was reported that a boosted regimen administering a dose on days 0 and 28 was necessary to maintain immunogenicity after 28 weeks and was not durable after 52 weeks [28]. An important follow-up study published in 2023 showed that immunogenicity could vary significantly between flavivirus-naïve and primed adults after administering ZIPV and that a third dose could not completely overcome this discrepancy [29].

Another inactivated whole-virus vaccine (TAK-426) with an aluminium hydroxide adjuvant was shown to be well tolerated and immunogenic in both flavivirus-naive and flavivirus-primed participants [30]. It was reported that the immune response after two intramuscular injections (days 0 and 28) in the 10 μg group was significantly greater than that in the 2 and 5 μg groups. A follow-up study showed that the 10 μg TAK-426 dose was durable up to 26 months with 100% neutralizing antibodies at one year, and 93.8 and 76.2% at 2 years in flavivirus-naive and flavivirus-primed groups, respectively [31]. Three DNA vaccines expressing premembrane and envelope ZIKV structural proteins were tested, of which two were published simultaneously: VRC5288 (Zika virus and Japanese encephalitis virus chimera) and VRC5283 (wild-type Zika virus) [32]. VRC5283 was generally well tolerated. As 14/14 individuals who received split-dose vaccinations by needle-free injection had detectable positive antibody responses, it was selected for further clinical development. The third DNA vaccine reported (GLS-5700) showed the presence of binding antibodies in all participants after a third intradermal dose delivered by means of electroporation and generated a protective response against multiple ZIKV isolates, although further studies are warranted, as the sample size was rather limited [33]. The prophylactic ZIKV vaccine Ad26.ZIKV.001, an adenovirus serotype 26 vector encoding ZIKV M-Env, was administered to healthy volunteers at a single lower (5 × 10^10^ vp) or higher (1 × 10^11^ vp) dose and appeared well tolerated [34]. Both lower and higher dosages showed potent antibody responses after a single dose and appeared durable after one year, especially with a boosted regimen. The authors reported that the vector vaccine could be a promising candidate for further development if the need reemerges, indicating that it has been shelved until a new epidemic/pandemic occurs. Interestingly, a follow-up study has recently reported that a single immunization with Ad26.M.Env ZIKV vaccine, when administered prior to conception, fully protects pregnant rhesus macaques from ZIKV viral RNA in blood and tissues, hinting towards further clinical development, which could also be of interest for (pregnant) travellers going to ZIKV-endemic areas [35]. Finally, in 2023, there was a report on the safety, tolerability, and immunogenicity of two mRNA vaccines (mRNA-1325 and mRNA-1893) [36]. Only mRNA-1893 (encoding the prME from the RIO-U1 Zika virus isolate) showed robust ZIKV-specific neutralizing antibodies after two doses on day 57, independent of flavivirus serostatus of the participants, and supported continuing clinical development.

Although success has been achieved with newer vaccine platforms, such as DNA and mRNA vaccines, alternative strategies are being developed, such as the manipulation of virus-resident immune microenvironments. In short, the authors described the ability to entrap a virus while limiting its release by using a virus-entrapping hydrogel that recruits and regulates the immune cells at the injection site, thereby controlling the timing and location of virus processing [37^▪▪^]. A single-dose vaccination prepared by loading live pathogenic ZIKV into the scaffold without prior treatment evoked effective immunity and protected the mice against lethal infection. Another approach is to generate herd immunity of wildlife hosts against ZIKV by releasing an insect-specific flavivirus (ISF) vectored vaccine via mosquito bites [38]. Recent studies have shown that mosquito-delivered vaccines can elicit robust immune responses against ZIKV in mice and conferred complete protection against ZIKV challenge, thereby interrupting the transmission cycle [39,40]. Although progress has been made with the development of several different vaccine platforms, all of which are generally well tolerable, only some show great potential for further development and are being shelved, as long as ZIKV circulation is too limited to allow planning and conduct of adequately powered efficacy trials.

### Zika virus immunogenicity and antibody applications

Previous cohort studies have shown that patients with ZVD do not always develop neutralizing antibodies, and the humoral immunity elicited is not long-lasting, which suggests that new flavivirus infections may occur within years after exposure [41]. Indeed, a recent study provided evidence of ZIKV reinfection [42]. Most studies have focused on IgG, as this is associated with neutralizing activity; however, a more recent study identified a potential role for IgM antibodies in protection against ZIKV *in vivo*[43]. Furthermore, when looking at the molecular level in patients with and without a robust antibody response, specific immune signatures were found and could predict antibody levels after infection [44]. In addition to the development of vaccines, antibody applications that neutralize ZIKV are of great interest. They may be used to disrupt the transmission cycle in endemic areas but could also be used as passive immunity for pregnant women or immunocompromised people travelling to endemic areas or may be used in therapeutic applications. In short, antibody-based immunotherapy can be divided into synthetically derived antibodies, such as mAbs, or convalescent plasma therapy (CPT), which are neutralizing antibodies derived from the plasma of patients or animals being infected [45]. Recent advances in mAb discovery techniques have allowed for the development of multiple therapeutic mAbs targeting ZIKV, although most are still in the preclinical development stage and have been extensively reviewed elsewhere [46]. In brief, mAbs generated to neutralize ZIKV target the envelope (E) protein, which is the major antigenic target in both ZIKV and dengue virus (DENV serotype 1–4). As both viruses show great similarity, ZIKV targeting antibodies could be cross-reactive with DENV. However, cross-reactivity at suboptimal concentrations or poor neutralization potency could potentially lead to antibody-dependent enhancement (ADE), a risk factor for the development of severe disease. Indeed, data gathered from a cohort study including 3412 children in Nicaragua showed that children with a previous ZIKV infection may be more vulnerable to symptomatic infections of certain types of DENV [47^▪^]. It was concluded that introducing a vaccine to protect against ZIKV may negatively affect dengue outcomes in individuals who have not yet had dengue. In line with this, a recent study suggested a potential negative impact of preexisting DENV immunity on subsequent ZIKV infection during pregnancy *in vivo*[48].

One way to overcome ADE is to develop ZIKV-specific neutralizing antibodies that target ZIKV-specific proteins without DENV overlap [49]. An alternative strategy is to develop a broadly neutralizing antibody targeting highly conserved sites within the NS1 epitope, which can achieve pan-flavivirus cross-protection and generate antibodies that are strongly immunogenic to all flaviviruses, including all DENV serotypes. Indeed, anti-NS1 also appears to protect against ZIKV replication in preclinical studies [50,51]. However, the details on which cross-reactive antibodies against ZIKV and DENV could protect, or mediate, pathogenesis are far more complex [52]. To date, clinical trials for the development of ZIKV targeting mAbs are limited and no phase 1 trials have been published [45]. Notably, a recent study showed that a single viral mutation can confer complete escape from targeted neutralizing antibodies, regardless of epitope, and therefore stresses the importance of examining the potential of these new therapies via deep mutational scanning and other techniques before clinical development [53^▪^].

The use of CPT is especially important as a therapeutic intervention, as severe clinical syndromes have been described for which no treatment exists, but also to neutralize the virus in pregnant patients, especially during their first trimester, to prevent potential microcephaly in the unborn foetus. In 2021, a phase 1 trial was published to study the safety, tolerability, and pharmacokinetics of human anti-ZIKV immunoglobulin (ZIKV-Ig; Emergent BioSolutions Inc., Gaithersburg, MD, USA) administered to healthy volunteers [intravenous (i.v.) dose (∼ 50–100 mg/kg)] [54]. ZIKV-Ig is a hyper-immune serum from pooled plasma of healthy donors with elevated antibodies reactive to ZIKV and appeared well tolerated. The pharmacokinetics (PK) parameters were consistent with the expected PK of other commercially available human Ig products, such as Hepatitis B Ig and Varicella Zoster Ig, from the same company. Although its clinical use has not yet been proven, the product has a calculated mean half-life of 28 days.

### Antivirals

Antivirals could be used for the rapid neutralization of ZIKV in cases where viremia is particularly unwanted, such as in pregnancy or in immunocompromised patients. Favipiravir and sofosbuvir both show antiviral activity but have only been tested in animals [55,56]. One group discovered two novel anti-ZIKV drugs by comparing viral infection temporal gene expression profiles to a drug-gene interaction database; however, these drugs have not yet entered clinical trials [57]. Only galidesivir, an adenosine nucleoside with a broad therapeutic range, including ZIKV, has undergone a phase 1 clinical trial and has been reported to be well tolerated [58].

## CHALLENGES AND FUTURE DIRECTIONS

The diagnostic differentiation of past DENV and ZIKV infections remains an obstacle for seroprevalence studies. The development and implementation of global wastewater surveillance tools appears to be one way forward to better predict epidemics. Further clinical development of antibody application and vaccines will be conducted when ZIKV circulation increases, as there are many products in the clinical pipeline that show great promise. It is especially important for women who wish to become pregnant or who are pregnant.

## CONCLUSION

While there is no current major ZIKV outbreak, progress has been made in the epidemiological work-up of clinical-epidemiological data, refinement of diagnostic tools, and mainly preventive (vaccines) rather than curative (drugs) tools.

## Acknowledgements

*None.*

*Invited review: Monkeypox and Emerging Diseases section for the March 2025 issue of Current Opinion in HIV and AIDS.*

### Financial support and sponsorship

*None received.*

### Conflicts of interest

*There are no conflicts of interest.*

## REFERENCES AND RECOMMENDED READING

Papers of particular interest, published within the annual period of review, have been highlighted as:

▪ of special interest
▪▪ of outstanding interest
